# Modeling Chemical Reaction Networks Using Neural Ordinary Differential Equations

**DOI:** 10.1021/acs.jcim.5c00296

**Published:** 2025-04-22

**Authors:** Anna C. M. Thöni, William E. Robinson, Yoram Bachrach, Wilhelm T. S. Huck, Tal Kachman

**Affiliations:** † Donders Centre for Cognition, Radboud University, Nijmegen 9103 6500 HD, The Netherlands; ‡ Institute for Molecules and Materials, Radboud University, Nijmegen 9010 6500 GL, The Netherlands; § Meta FAIR, London N1C 4DB, United Kingdom

## Abstract

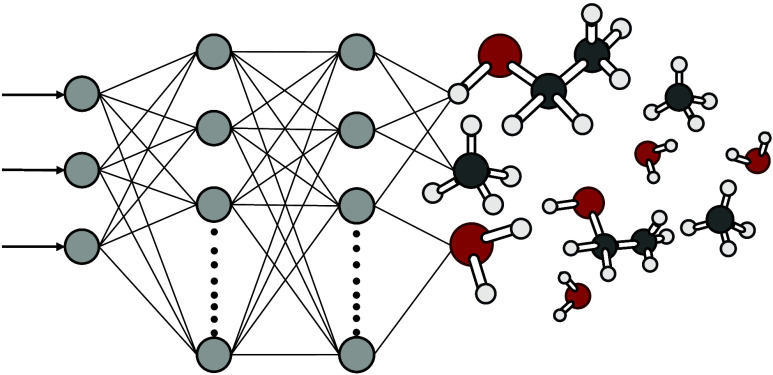

In chemical reaction network theory, ordinary differential equations are used to model the temporal change of chemical species concentration. As the functional form of these ordinary differential equation systems is derived from an empirical model of the reaction network, it may be incomplete. Our approach aims to elucidate these hidden insights in the reaction network by combining dynamic modeling with deep learning in the form of neural ordinary differential equations. Our contributions not only help to identify the shortcomings of existing empirical models but also assist the design of future reaction networks.

## Introduction

The dynamic behavior of networks of chemical reactions is typically described using a system of ordinary differential equations (ODEs). Such systems of ODEs are derived by combining the chemical reaction network (CRN), which underlies the observed dynamics, with the law of mass action.^1−3^ The parameters of the ODEs, such as the rate constant of a reaction or the concentration of a reactant, are ideally experimentally determined or need to be calibrated to maximize the similarity between the model predictions and the experimental data over time.^4,5^

Despite this calibration, it is not guaranteed that the ODEs can predict the experimental data perfectly, as the data can be noisy or incomplete.^6^ The proposition of mass action assumes that the reaction mixture is well-mixed and under thermal equilibrium but these requirements are not always fulfilled in practice. Furthermore, the differential equations cannot describe any dynamics that are not included in the theoretical specification of the CRN. This limits the modeling performance when the CRN is misspecified, for example, by the presence of hidden interactions or competition between the species involved.^7,8^ The limited theoretical modeling performance may hamper the quality of the predicted behavior of chemical systems consisting of multiple reactions.

We propose to improve the predictive behavior of ODEs by combining dynamic systems modeling with deep learning in the form of Neural ODEs (nODEs).^9^ nODEs are akin to deep learning models,^10^ which drive many recent successes, from self-driving cars,^11^ through large language models^12,13^ to protein structure prediction.^14^ Deep learning models use artificial neural networks, mathematical models that can be trained to approximate any function.^15^ However, nODEs are inspired by the advances in deep residual learning and normalizing flows, which build complex transformations by applying a sequence of smaller transformations;^16,17^ unlike standard deep learning models that operate through a discrete sequence of hidden layers, nODEs employ a neural network to *parametrize the derivative of a hidden state*. As such, the nODE output is computed through a differential equation solver. Thus, in nODEs, neural networks represent the smaller transformations, and in the limit, the composition of small neural transformations behaves like a differential equation.^9^ However, in contrast with a predefined differential equation, the nODE takes form during training,^18^ allowing the nODE to minimize the difference between the observed and modeled data. Therefore, we hypothesize that the predictions of the nODEs are of a better quality than those from the original theoretical model.

The possibility of solving chemical kinetics using nODEs has been explored before. Nonetheless, the key limitations of these earlier methods are that they require that the number of reactions is known a priori^19^ or focus on the thermodynamic state of the reactions.^20^ In contrast, we aim to address the differences between the modeled and experimental concentrations *without making any assumptions about the reactions within the CRN*. To this end, we follow Rackauckas et al.^21^ and augment the existing theoretical system of ODEs with a neural network component. The neural network acts as a correction term: it captures the trends within the experimental data that the theoretical model does not explain. The network contribution can be calculated for each measured chemical species. The separate contributions provide insight into the magnitude and temporal signature of the differences between the theoretical model and the experimental data.

We test our methods by predicting the phase space of an oscillatory system. We focus on oscillating behavior as the quality of the model prediction is easily jeopardized by hidden interactions between the species involved. Predicting the amplitude, periodicity, and phase space accurately is challenging as the model needs to account for noisy measurements, hidden interactions, and dynamics that are out of equilibrium. Therefore, we split the prediction of the oscillator dynamics into smaller objectives. We first consider two experimental data sets collected by ter Harmsel et al.,^22^ who composed a chemical oscillator of reactions between small organic molecules. The CRN consists of four reactions and involves 7 species. The reactions include the autocatalytic Fmoc-piperidine (2) deprotection via dibenzofulvene (6); the *N*-methylpiperidine-catalyzed (5) Fmoc-piperidine deprotection; the fast inhibition via acetylation by *p*-nitrophenyl acetate (3); and the slow inhibition by phenyl acetate (4) that converts piperidine (1) to N-acetyl piperidine (7).^22^ We use the data from the single-pulse (aperiodic, batch) experiment as well as the measured series of sustained oscillations, which require an open system (flow reactor). Furthermore, we test the model’s performance on hidden interactions by deliberately leaving out reactions from the CRN described above. Finally, we predict the oscillation periodicity and space of a CRN by transferring the dynamics captured by the nODE between experiments.

The results found provide two insights. First, the neural network contributions can be used to verify the correctness of the theoretical model under noisy measurements. Second, the contributions can be used to account for and uncover any dynamics that are not included in the theoretical model. Combining these findings, we show that nODEs are more accurate predictors of the period of oscillating concentrations of unseen experimental settings than the theoretical system of ODEs alone.

## Methods

### Chemical Reaction Networks and nODEs

Before introducing our nODE approach, we briefly discuss dynamical systems modeling with ODEs. To do so, we consider modeling the concentration of chemical species by solving the initial value problem (IVP). More formally, let the dynamical system be defined from time *t*_0_ until *T*, where *y* describes the unknown solution represented by the initial point *y*_0_ and *h*(*t*, *y*(*t*)) a system of ODEs that act as a constraint on the solution. With these terms, the IVP is defined in eq 1.
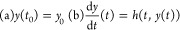
1While the solution to eq 1 equals *y* = ∫_*t*_0__^*T*^*h*(*t*, *y*(*t*))d*t*, systems of differential equations are often solved using numerical methods due to the unavailability of the antiderivative of *h*.^23^ Numerical methods calculate the full time-series *y* step-by-step, starting at *y*_0_, and going to *y*(*T*) with small increments of *δt*. When CRNs are considered, *h* represents the theoretical system of ODEs, the mathematical description derived from the CRN that satisfies the law of mass action. *y* describes the concentrations of the chemical species over time, *y*_0_, the initial concentration, and  the change in concentration over time *t* ∈ [*t*_0_, *T*].

In nODEs, and universal differential equations in particular, the existing theoretical system of ODEs (eq 1b) is augmented with the data-driven neural network *f*_θ_.^21^ As a consequence, the description of the chemical kinetics is no longer predefined. Instead, it has become data-driven, where *f*_θ_ is trained to minimize the difference between the nODE predictions and the experimental observations. A more formal description of the nODE, relating it to the constraint on the IVP presented in eq 1b, is provided in eq 2.
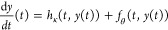
2Here, κ represents the parameters of *h*, in our case the vector of reaction rate coefficients *k*_*tr*_, *k*_*ac*_, *k*_*inh1*_ and *k*_*inh2*_, retrieved from ter Harmsel et al.,^22^*h* represents the full system of ODEs, describing the rate of change of each chemical species in the oscillating CRN (eq 3). The concentration of *N*-methylpiperidine (5) stays constant over time and is therefore not included. The species are abbreviated with their index on the right-hand side of the equation.
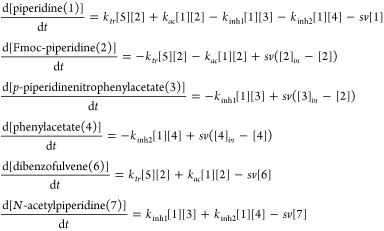
3The stepsize *dt* is governed by the differential equation solver. We use Kværnø’s  method to account for the stiffness of the ODE.^18,24^ Furthermore, we change the temporal scale of the experimental data from seconds to hours (single-pulse) or days (oscillations) to reduce the total number of steps the solver makes.

### Neural Network Architecture

The neural network used consists of an LSTM cell with a 32-dimensional hidden state followed by a linear layer of 32 neurons. The network uses identity activations before and after the application of the linear layer. The number of inputs and outputs of the network matches the number of species that have been measured during the experiments. At each step within the equation solve, the neural network is presented with the measurements at a single time point instead of the full time series. Still, the LSTM outperforms a fully connected neural network during the experiments with synthetic data (see the Supporting Information). The model has been implemented using JAX^25^ version 0.4.14 and Equinox^26^ version 0.11.1.

### Neural ODE Training and Inference

Using eq 2 and the initial concentrations of each species, we train *f*_θ_ to match the experimental data at the measurement times *t*. The network parameters θ are updated according to a mean squared error (MSE) loss with respect to the species that have been measured. Our data consists of the mean and standard deviation calculated over two experimental measurements. We generate artificial training, validation, and test data by drawing from a normal distribution that is parametrized with these statistics. The neural network has been trained on *N* = 1000 train samples using an Adabelief optimizer with a learning rate of 6 × 10^–3^.^25,27^ We did not apply dropout as this was found to cause instabilities during the differential equation solve, which resulted in a large number of rejected solver steps. For the oscillating data, we first train the nODE on the first 10% of each time series to prevent getting stuck in a local minimum.^18^ Next to the nODE predictions, we calculate the separate neural network contribution  given the prediction of the complete differential equation .
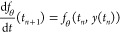
4

### The Identification of Missing Reactions

We use a similar modeling approach for the identification of missing reactions. However, instead of subtracting a constant concentration for each time period, we remove a part of the theoretical system of ODEs. In particular, we modify the change in N-acetyl piperidine to the definition presented in eq 5. This modification partially removes the pathway of the second inhibition reaction of the enzymatic oscillator. The rest of the theoretical system of ODEs remains unaltered.
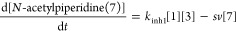
5

## Results and Discussion

### Modeling Experimental Data using nODEs

**Figure 1:**
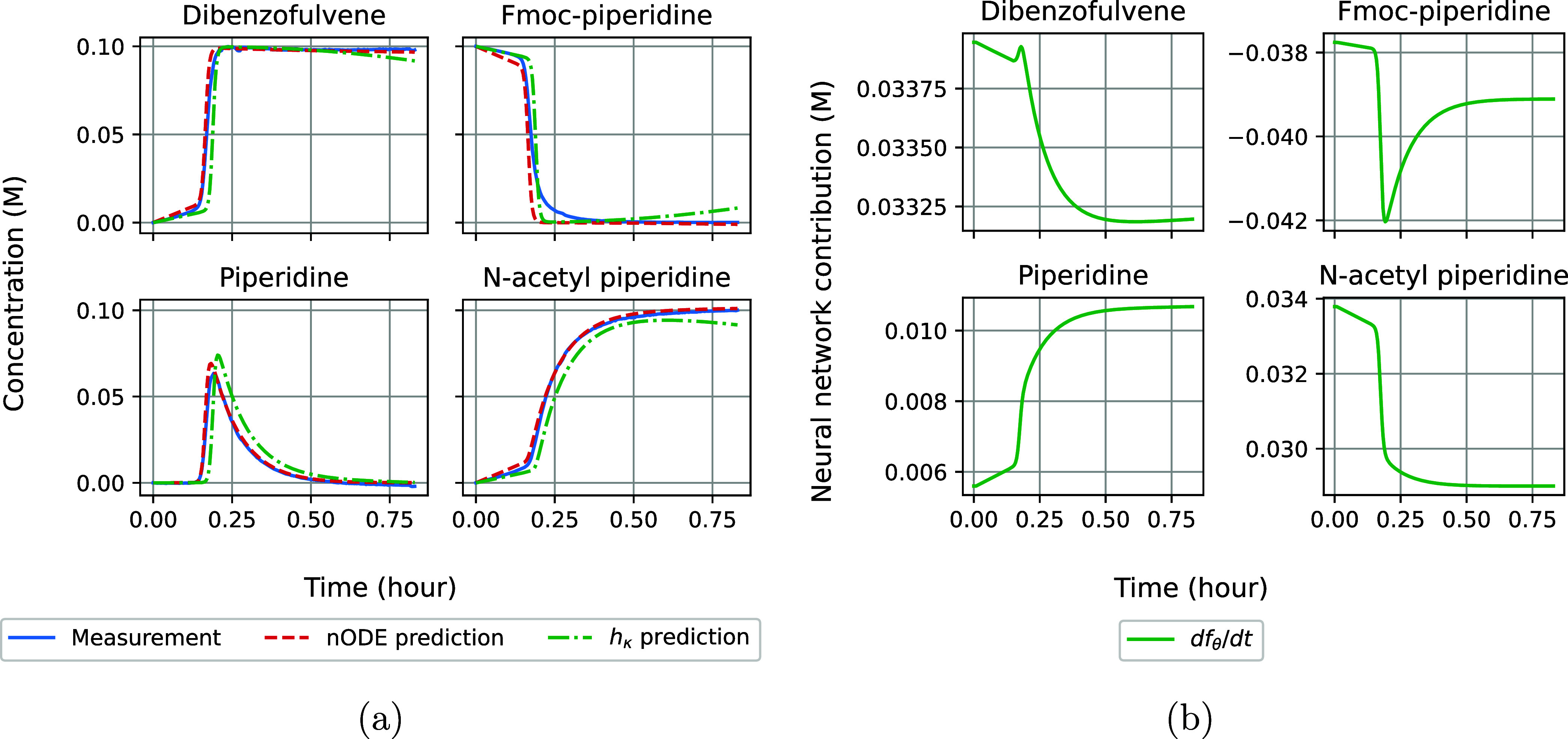
Predictive performance of the nODE on the single-pulse data. (a) The experimental measurements (solid blue), predictions from the nODE (dashed red), and *h*_κ_ (dashdotted green). The four panes illustrate the molar concentrations for the species dibenzofulvene, Fmoc-piperidine, piperidine, and N-acetyl piperidine. (b) The neural network contribution for the four measured species.

We first consider the performance of the ”standard” ODE: the system of differential equations that only includes *h*_κ_ as described in eq 3. Figure 1 illustrates the results for the single-pulse experiment based on this model. The experimental measurements (solid blue), nODE predictions (dashed red), and the standard predictions based on *h*_κ_ (dashdotted green) for the species Fmoc-piperidine, dibenzofulvene, piperidine, and N-acetyl piperidine are shown in Figure 1a. The figure shows that the nODE, combining *h*_κ_ and *f*_θ_, provides a better fit than *h*_κ_ alone. This relative improvement can be attributed to the positive neural network contributions for the species dibenzofulvene and piperidine and N-acetyl piperidine, and the negative contribution for Fmoc-piperidine (Figure 1b). The neural network contributions change over time. The rate of their change is the strongest at the start of the reaction and tends to flatten toward the end, indicating convergence toward a steady state.

#### Compensating for Hidden Interactions

**Figure 2:**
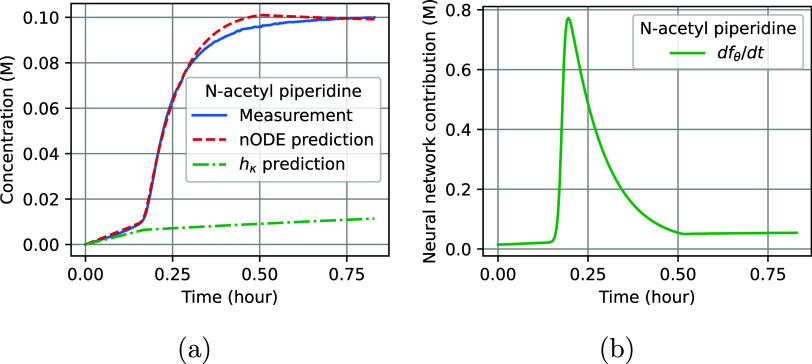
Predictive performance of the nODE for which the reaction for N-acetyl piperidine is hidden. (a) The experimental measurements (solid blue), predictions from the nODE (dashed red), and *h*_κ_ (dashdotted green) for N-acetyl piperidine. (b) The neural network contribution.

We demonstrate the resilience of the nODE against hidden interactions that are not included in the theoretical model of the CRN. To this end, we adapt the theoretical model *h*_κ_ by removing the slow inhibition pathway via phenyl acetate, which negatively affects the creation of N-acetyl piperidine. We assess the modeling performance under this condition by focusing on the concentrations of N-acetyl piperidine, shown in Figure 2. Even though the nODE does not provide a perfect fit (Figure 2a), the missing variable is largely accounted for by an increase in the neural network contribution in Figure 2b compared to Figure 1b. The predicted concentrations of the remaining species can be found in the Supporting Information.

#### Oscillating Reaction Networks

**Figure 3:**
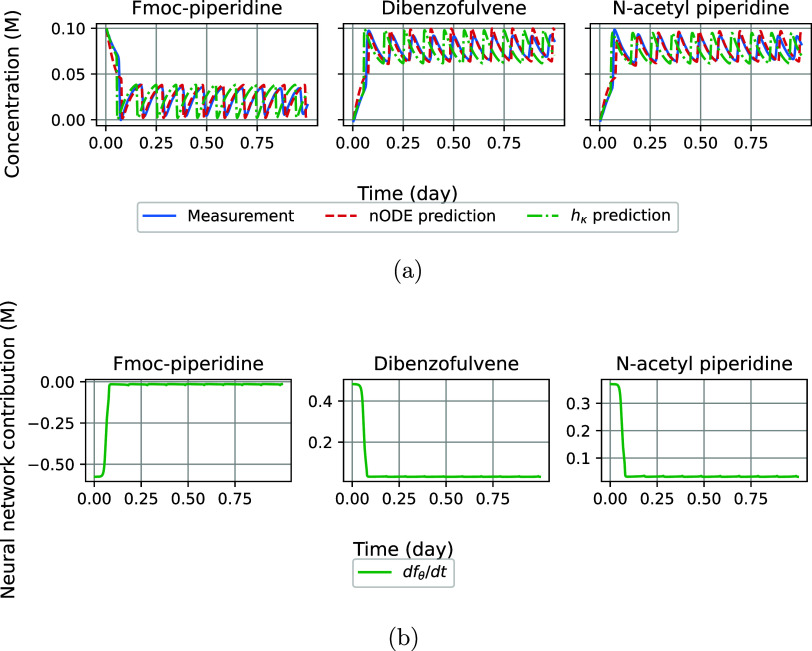
Predictive performance of the nODE on the oscillating open system for dibenzofulvene. (a) The experimental measurements (solid blue), predictions from the nODE (dashed red), and *h*_κ_ (dashdotted green). (b) The neural network contribution.

Lastly, we evaluate the predictive performance of the nODE on the oscillating CRN. The results of this experiment are shown in Figure 3. When comparing the predictions of the nODE and *h*_κ_ in Figure 3a, it becomes clear that the oscillations within the data have a different frequency than those predicted by *h*_κ_. The neural network contribution (Figure 3b) ensures that the oscillations predicted by the nODE are more time-locked with the data. This effect is particularly strong at the start of the reaction, as the data and the predictions tend to go out of phase later. As the observed phase changes over time, the improved time-keeping ability of the nODE compared to *h*_κ_ demonstrates that these changes can be approximated with a neural network.

#### Interpreting the Neural Network Contribution

As demonstrated above, the data-driven contributions from the neural network *f*_θ_ set the nODEs apart from the theory-based ODEs. The refinements provided by the nODE can be leveraged in two ways. First, the learned contributions provide insights into the magnitude and temporal signature of the discrepancies between the theoretical model and the data. To connect these new insights with the theoretical model, the neural network contributions may be translated into concrete mathematical expressions via symbolic regression,^28^ ultimately improving our understanding of the relations between the compounds included in the CRN. Nonetheless, the neural network contributions may be hard to interpret: not only do they capture any missing dynamics, but they also aggregate over all forms of noise within the system. Therefore, a second use case of the nODEs focuses on transferring the learned dynamics between experiments. Doing so, we assume that the noise around the observations is independent and identically distributed (iid.) across experimental settings. While this may be a strong assumption, it is partially fulfilled as long as the experiments are carried out under the same conditions. Transferring information between experimental settings can be beneficial when predicting experimental outcomes. The next section explores this idea in the context of oscillating CRNs.

### Predicting the Oscillation Space

In oscillating CRNs, sustained oscillations are only observed for a relatively narrow set of input values for the control parameters. Outside this regime, the system can either show a steady state or damped oscillations that decay toward a steady state. The concentrations of species flowing into the reactor must therefore be chosen carefully. This is a major experimental challenge, as the system of ODEs often does not predict the oscillatory regime quantitatively correct, and experiments are laborious and time-consuming. We use the nODE to predict the regime of sustained oscillations for various concentrations of Fmoc-piperidine and phenyl acetate that are supplied in flow. As these compounds are part of the positive and negative feedback loops within the CRN, any variation of their input values affects the phase and stability of the oscillations. As a consequence, the oscillation space of the experiment is described by the Cartesian product of the different concentrations in flow. It is important to note that we do not train a nODE for each experimental setting. Instead, we train nODE once and transfer the learned dynamics between experiments.

**Figure 4:**
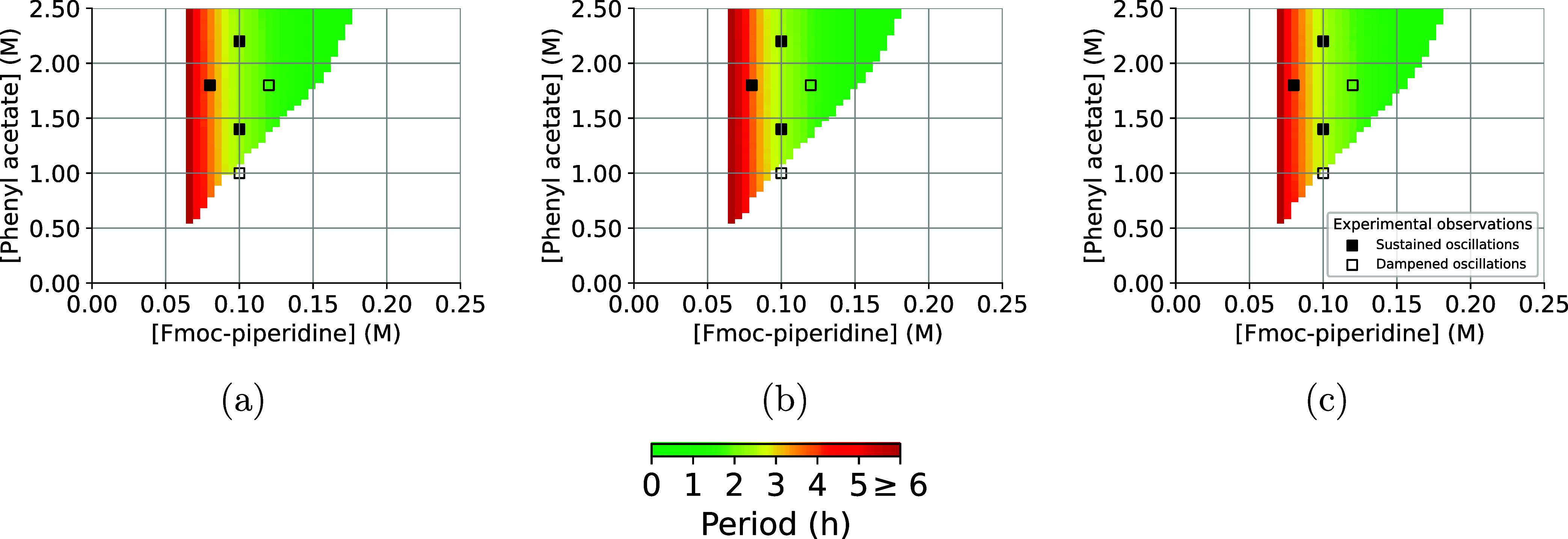
Oscillation spaces predicted by (a) the theoretical model, (b) the nODE trained on the complete time series, and (c) the nODE trained on the first half of the time series. The colored regions show the predicted period of the oscillations. Outside of these regions, the models predict damped or no oscillations. The filled squares indicate experimentally observed sustained oscillations, the open squares are associated with damped oscillations.^22^

The predictions of the theoretical model, a nODE trained on the first half of the oscillations and a nODE trained on all oscillations are shown in Figure 4. The models are trained on data from the experimental setting where 100 mM Fmoc-piperidine and 1.8 M phenyl acetate are presented in flow. Once the models are trained, they are presented with different concentrations of phenyl acetate and Fmoc piperidine in flow, spanning the oscillation space. The predicted oscillation spaces are compared to experimental observations, indicated with (filled) squares. The area in which sustained oscillations are predicted does not differ greatly between the models: all models make a correct prediction for four out of five observed oscillations.

The fifth experiment, where 120 mM Fmoc-piperidine and 1.8 M phenyl acetate are supplied in flow, is incorrectly predicted as a source of stable oscillations. Even after having trained on the data collected during all five experiments, the nODE was not able to correctly adjust the regime of stable oscillations. This limitation could be fueled by a variable initialization time between experimental setups, violating the assumption that the noise is iid. between experiments. For example, it is possible that the chemicals are not instantaneously well-mixed in the continuous stirred-tank reactor (CSTR), which is an important assumption underlying all work in such reactors. Breaking this fundamental assumption would introduce a large discrepancy between the theoretical model and the measured data. If the initialization time varies between experiments, then the nODE may learn spurious relationships that inhibit the generalization between stable and damped oscillations. When the latter is combined with the small number of experiments under consideration, it can be very hard to learn transitions between stable and decaying oscillations.

**Figure 5:**
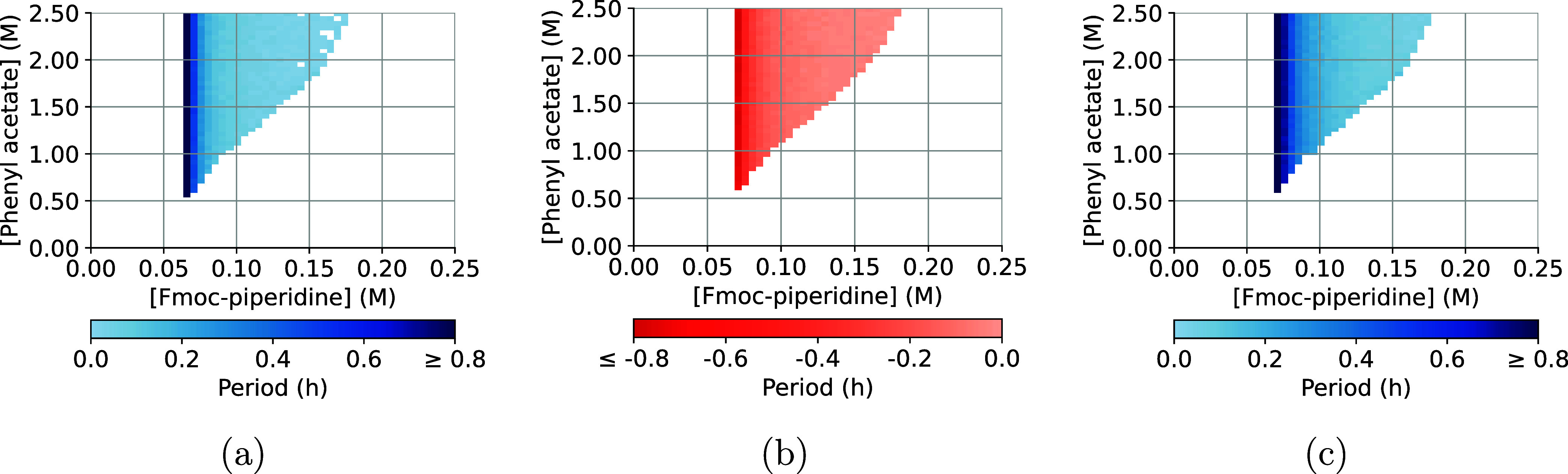
Difference between the predicted oscillation spaces. (a) The predictions of the nODE trained on the full time series minus the theoretical predictions. (b) The predictions of the nODE trained on the full time series minus those of the nODE trained on half of the time series. (c) The predictions of the nODE trained on half of the time series minus the theoretical predictions.

While the nODEs are no better predictors of the type of oscillation than the theoretical model, they provide a better estimate of the oscillation period. As highlighted in Figure 5, the predicted oscillation spaces differ in the size of the periods. In particular, the periods predicted by the nODE trained on the first half of the time series generally predict the largest periods, followed by the nODE trained on the full time series and the theoretical model.

The trend found in Figure 5 recurs in Table 1: the nODE-based models tend to predict larger periods than the theoretical model (abbreviated with ODE). As a result, the periods predicted by the nODEs are a more accurate representation of the experimentally observed period than the predictions based on the theory alone.

**Table 1:** Observed and Predicted Periods of Different Experimental Settings

experimental setting		observed period	ODE	nODE	nODE half
[Fmoc-piperidine] (M)	[Phenyl acetate] (M)	(h)	(h)	(h)	(h)
0.08	1.8	4.3	3.52	3.70	4.00
0.1	1.4	2.7	2.37	2.45	2.55
0.1	2.2	3.6	2.33	2.40	2.52

## Conclusions

The real-world behavior of chemical species during a reaction can be modeled with a system of ODEs. The quality of this model is affected by the theoretical models that describe the chemical system, which can be incomplete. To account for the differences between the experimental data and the theoretical model, we combine dynamic systems modeling and deep learning in the form of neural ODEs.

Our results demonstrate the applicability of a nODE for modeling aperiodic and oscillating concentrations. The separate neural network contributions provide an insight into the nature of the residuals between the theoretical model and the data.

The neural network contributions may be hard to interpret, as they aggregate over all forms of noise within the system. Therefore, we focused on predicting the periodicity and phase space of an oscillating CRN by transferring the dynamics learned by the network between experiments. The nODEs do not outperform the theoretical ODE when classifying the regime of sustained oscillations. Despite training on experiments that represent various locations within the phase diagram, the neural network is unable to learn the dynamics of the transition between stable and decaying oscillations. We believe that additional experiments would be necessary in order to provide more training data that capture the deviations from ideal behavior. Nonetheless, the nODEs are a valuable addition to the theoretical model, as they are a better predictor of the period of the oscillations. We advocate training the nODEs in conjunction with the development of the experimental systems and use the nODE predictions as a guide to which additional experiments would provide maximum improvement in nODE prediction. Altogether, we expect nODE predictions to help identify shortcomings of existing theoretical models and assist in the future design of functional CRNs.

## Data Availability

The source code associated with the generation of synthetic data and the methods presented is available on GitHub: https://github.com/KachmanLab/ChemicalReactionNetworks. All software was prepared by the Radboud University authors. The experimental data used in this work have been publicly made available by ter Harmsel et al. and can be found online: https://www.nature.com/articles/s41586-023-06310-2#Sec4.^22^

## Supporting Information Available

Extra information on neural networks, chemical reaction networks, and modeling dynamical systems; pseudocode of the algorithms presented in the methods; and the additional experimental results considering experimental data (PDF)
