# Supplementary material for: Modeling Chemical Reaction Networks Using Neural Ordinary Differential Equations
Source: J Chem Inf Model. 2025 Apr 22;65(9):4346–52. doi: 10.1021/acs.jcim.5c00296 (PMC12076499; doi:10.1021/acs.jcim.5c00296)
Supplement: Supplementary file 1 — ci5c00296_si_001.pdf [file ci5c00296_si_001.pdf]

Supporting Information: Modeling Chemical Reaction Networks using Neural Ordinary Differential Equations

Anna C. M. Thöni,[*,†] William E. Robinson,[‡] Yoram Bachrach,[¶] Wilhelm T. S. Huck,[‡] and Tal Kachman[†]

†*Donders Centre for Cognition, Radboud University, Nijmegen 9103 6500 HD, the Netherlands*

‡*Institute for Molecules and Materials, Radboud University, Nijmegen 9010 6500 GL, the Netherlands*

¶*Meta FAIR, London N1C 4DB, United Kingdom*

E-mail: chiara.thoeni@ru.nl

Background

Artificial Neural Networks

A neural network is a universal approximator that consists of the repeated application of layers. Each layer combines a linear and nonlinear transformation $\mathbb{R}^D \to \mathbb{R}^M$. In a multilayer perceptron, a popular architecture, the output of the jth layer, where $j \in 1, ..., M$, is described by eq 1.[S1]

$$a_j = b\left(\sum_{i=1}^{D} w_{ji}x_i + w_{j0}\right) \tag{1}$$

where w_{ji} represents the weight that is applied to the input x_i and w_{j0} is the bias. b is a nonlinear, layer-specific activation function. The architecture of the network, i.e., the number of layers, their widths D and activation functions b, is determined prior to the training process. In contrast, the values of the weights w are learned from the training data.

Network Training The data needed to train a neural network consists of a set of inputs $\{x_i\}$ with $i \in 1, ..., N$ and their corresponding labels y_i. The goal of the training process is to find the parameters θ that minimize the error function between the prediction of the network $f_\theta(x_i)$ and the labels y_i. In this work, we use the mean squared error (MSE) to calculate the loss of the network.

$$E = \frac{1}{N} \sum_{i=1}^{N} (f_\theta(x_i) - y_i)^2 \tag{2}$$

After each forward pass $f_\theta(x_i)$, the values of the weights are updated in the direction of the negative gradient that is associated with the error function. The magnitude of this update is regulated by the learning rate.[S1]

Recurrent Neural Networks The information flow within the MLP described above is unidirectional: the outcomes $\boldsymbol{a}$ are only propagated to the subsequent layers. Alternatively, the information flow can be bidirectional, where the outcomes of a layer are added to subsequent inputs to the same layer. As such, the networks are provided with a sort of "memory component". Within the class of these recurrent neural networks, the long short-term memory (LSTM) is a popular architecture.[S2]

Chemical Reaction Networks

A chemical reaction network (CRN) is a framework that describes the relationships between chemical reactions.[S3] To illustrate, consider the theoretical reaction network $A \rightarrow B \rightarrow C$,

consisting of the reactions $\mathcal{R} = \{R_1, R_2\}$:

$$\begin{aligned} R_1\colon\ & A \xrightarrow{k_1} B \\ R_2\colon\ & B \xrightarrow{k_2} C \end{aligned} \tag{3}$$

In this process, a single unit of compound A is converted to compound B with a reaction rate k_1. Next, compound B goes to compound C with rate k_2. The three different compounds that take part in the reaction are referred to as the species, $\mathcal{S} = \{A,\ B,\ C\}$ with $n_s = |\mathcal{S}| = 3$. In chemical reactions, the species on the left-hand side of the arrow are considered the reactants, whereas the species on the right-hand side are considered the products.

Dynamical Modeling and mass action Kinetics Using a CRN and the relations between species that it describes, one can construct a system of ODEs to determine the rate of concentration change of the compounds involved. More specifically, if the mixture of compounds is well-stirred and the number of compounds is sufficiently high, it can be assumed that the reactions $\mathcal{R}$ occur according to an exponential probability distribution.[S3,S4] As such, the rate r_i of each reaction $R_i \in \mathcal{R}$ can be described according to the mass action kinetics:

$$r_i(S) = k_i \prod_{j=1}^{n_s} [S_j]^{a_{ij}} \tag{4}$$

In eq 4, k_i is the reaction rate coefficient,[1] S is the vector of all species, $[S_j]$ is the concentration of species S_j and a_{ij} is the stoichiometric coefficient of species S_j in reaction R_i. The rate coefficient k_i can be determined by experiment or simulation.[S4]

Now, let C_i be the reactants and C_i' the products of R_i. Using this definition, we can determine the state change of R_i: if reaction R_i can be described by $C_i \to C_i'$, the state change can be summarized by $a_i \to a_i'$. Here, a_i is the vector of the stoichiometry coefficients of the set of reactants or products from reaction R_i. a_i is grouped by the species. For example, for

[1]In this work, the reaction rate coefficient is regarded as a constant as it is independent of the time t,[S5] the main control variable used.

R_1 it holds that $a_1 = [1, 0, 0]^T$ and $a'_1 = [0, 1, 0]^T$. Using the state change, the rate of the concentration change that is brought about by R_i is defined as the state change times the rate r_i, which results in a vector of n_s derivatives (eq 5).

$$\frac{d[S]}{dt} = (a'_i - a_i) \times k_i \prod_{j=1}^{n_s} [S_j]^{a_{ij}} \tag{5}$$

For CRNs with multiple reactions, the rate of change in the total concentration of a species is provided by the sum over all reactions $R_i \in \mathcal{R}$. Thus, returning to the CRN from eq a final time, if the reaction rates are assumed to be k_1 and k_2, one can describe the rate of the change in concentration of the three species as in eq 6.

$$\begin{aligned} \frac{d[A]}{dt} &= -k_1[A] \\ \frac{d[B]}{dt} &= k_1[A] - k_2[B] \\ \frac{d[C]}{dt} &= k_2[B] \end{aligned} \tag{6}$$

The vector field described by eq 6 can, in combination with the initial concentrations of each species, be used to obtain an estimate of the concentrations over time by solving the initial value problem (IVP).

Modeling Dynamical Systems

Expressing the Initial Value Problem in Neural Vector Fields. To provide an introduction to nODEs, we first define the IVP in more formal terms. Let y: $[0, T] \to \mathbb{R}^m$ be the unknown solution described by the initial point $y_0 \in \mathbb{R}^m$ and differential equation $h(t, y(t))$: $\mathbb{R} \times \mathbb{R}^m \to \mathbb{R}^m$. Using these terms, the IVP is formalized in eq 7.

$$y(0) = y_0 \qquad \frac{dy}{dt}(t) = h(t, y(t)) \tag{7}$$

While the solution to eq 7 equals $y = \int_0^T h(t, y(t))$, systems of differential equations are often solved using numerical methods due to the unavailability of the antiderivative of h.[S6] The simplest method to solve an IVP is to discretize the derivative according to the Euler discretization.[S7]

$$\frac{dy}{dt}(t_n) \approx \frac{y(t_{n+1}) - y(t_n)}{\Delta t} \tag{8}$$

Using the discretization above, the vector field from eq 7 can be rewritten to

$$y(t_{n+1}) = y(t_n) + \Delta t \cdot h(t, y(t_n)) \tag{9}$$

If h is represented by the neural network f_θ, then Δt can be absorbed into the neural network term to yield eq 10.[S8]

$$y(t_{n+1}) = y(t_n) + f_\theta(t_n, y(t_n)) \tag{10}$$

Chen et al. point out that eq 10 bears similarities with the formulation of a residual neural network,[S10] where the addition of $y(t_n)$ can be interpreted as the skip connections within the residual block. Fuelled by this observation, Chen et al. introduce neural ODEs, which they consider a residual network with a “continuous depth".[S9]

Universal Differential Equations Rackauckas et al. extend the nODEs by incorporating domain knowledge into the model. Their proposed universal differential equation combines the fully data-driven with a theoretical vector field h that is specified beforehand.[S11]

$$\frac{dy}{dt}(t_{n+1}) = h_\kappa(t, y(t_n)) + f_\theta(t, y(t_n)) \tag{11}$$

In eq 11, κ represents the parameters of h. When vector fields based on CRNs are considered, then h represents the vector field based on the mass action dynamics of the CRN. In that case, κ denotes the vector of reaction rate coefficients $\boldsymbol{k}$.

Differential Equation Solvers and Stiffness Solving a system of differential equations in a stepwise fashion as described above introduces a limitation: the performance of the ODE solvers may suffer from the stiffness of the IVP at hand. That is, IVPs that contain regions of strongly increasing or decreasing dynamics, an often-occurring phenomenon within chemical kinetics, can cause numerical instability Aro. Whereas Ji and Deng[S13] used soft constraints in their physics-informed neural network to alleviate this instability, it can be countered within nODEs by using an implicit ODE solver.[S14]

Pseudocode

Algorithm 1 Neural ODE training

Input

N_{epochs}: the number of epochs
T: the time of the last measurement
$\mathcal{D}$: the dataset $\{N \times (t_0,\, \boldsymbol{y}_0),\, (t_1,\, \boldsymbol{y}_1),\, \ldots,\, (T,\, \boldsymbol{y}_T)\}$
f_θ: the neural network with parameters θ
$h_{\boldsymbol{\kappa}}$: the theoretical system of ODEs

Output

The trained model f_θ

```
for epoch in N_epochs do
    for x, y in D do
        t_s ← t_0
        while t_s < T do
            if t_s is t_0 then
                ŷ_{t_s} ← y_0
            end if
            Δt ← ODEsolver(ŷ_{t_s}, t_s)
            ŷ_{t_s+Δt} ← h_κ(ŷ_{t_s}, Δt) + f_θ(ŷ_{t_s}, Δt)
            t_s ← t_s + Δt
        end while
        L = MSE(ŷ_{t_0:T}, y)
        Update θ by taking a gradient descent step on L
    end for
end for
```

Algorithm 2 Neural network contribution

Input

$\hat{\boldsymbol{y}}$: the concentrations predicted by the neural ODE.
f_θ: the neural network with parameters θ

Output

c_{f_θ}: the neural network contribution

```
for ŷ_t in ŷ do
    c_{f_θ,t} ← f_θ(ŷ_t)
end for
```

Supplementary Figures

This section presents all results that have been found when considering the experimental data, summarizing the standard fit, open system and missing reaction experiments with the single-pulse and oscillating data. The section serves as a reference without providing an in-depth description of all figures. Instead, all conclusions drawn from these results are provided in the main document.

Single-pulse Experiment

Standard Fit

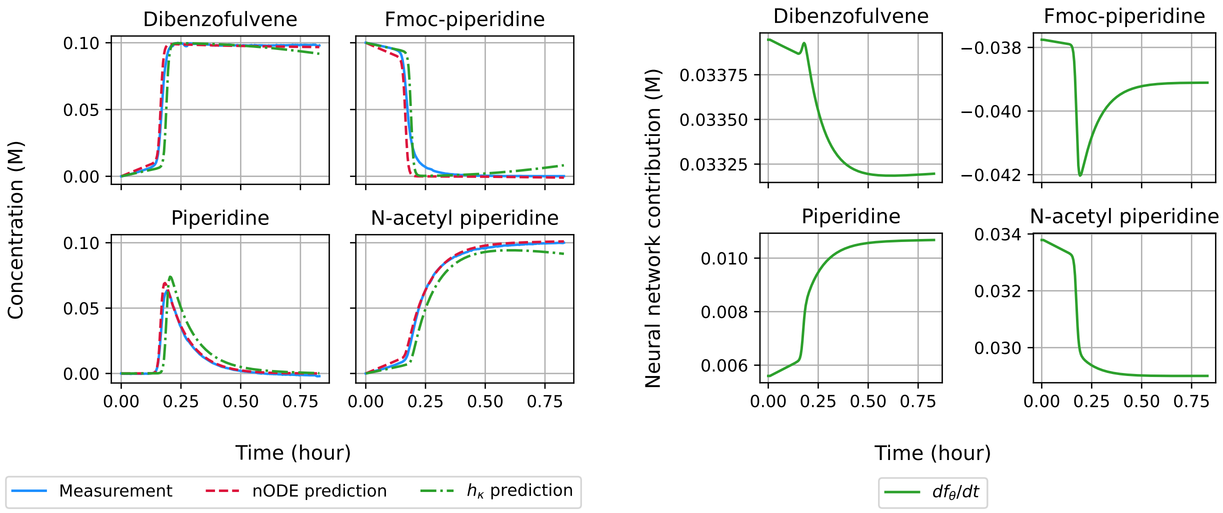

(a) (b)

Figure S1: Predictive performance of the nODE on the single-pulse data. (a), The experimental measurements, predictions (solid blue) from the nODE (dashed red) and h_κ (dashdotted green). The four panes illustrate the molar concentrations for dibenzofulvene, Fmoc-piperidine, piperidine, and N-acetyl piperidine. (b), The neural network contribution for the four measured species.

Open System

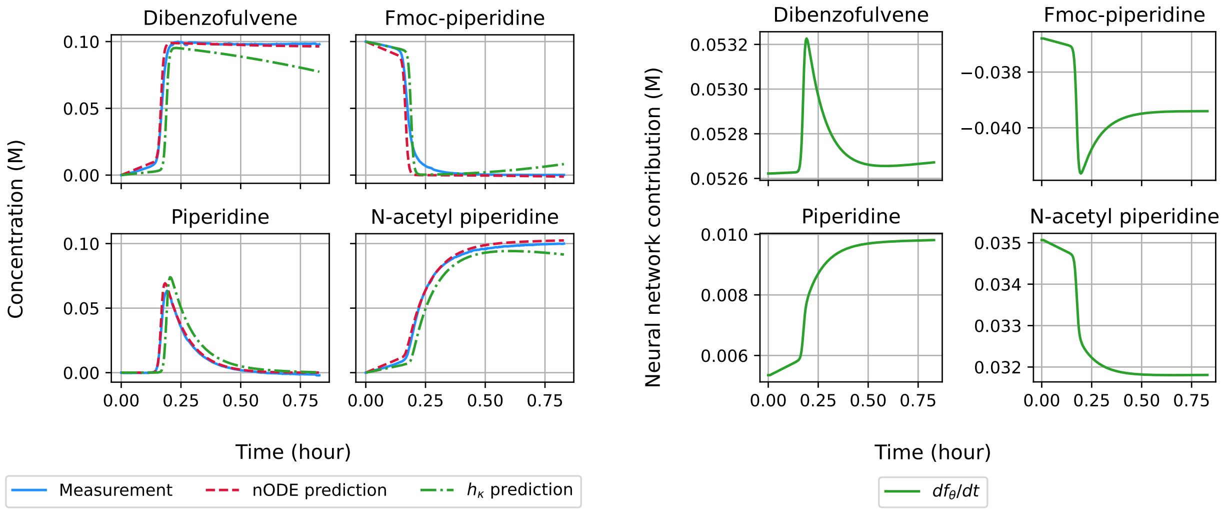

(a) (b)

Figure S2: Predictive performance of the nODE on the single-pulse data in the open system. (a), The experimental measurements, predictions (solid blue) from the nODE (dashed red) and h_κ (dashdotted green). The four panes illustrate the molar concentrations for the species dibenzofulvene, Fmoc-piperidine, piperidine, and N-acetyl piperidine. (b), The neural network contribution for the four measured species.

Missing Reactions

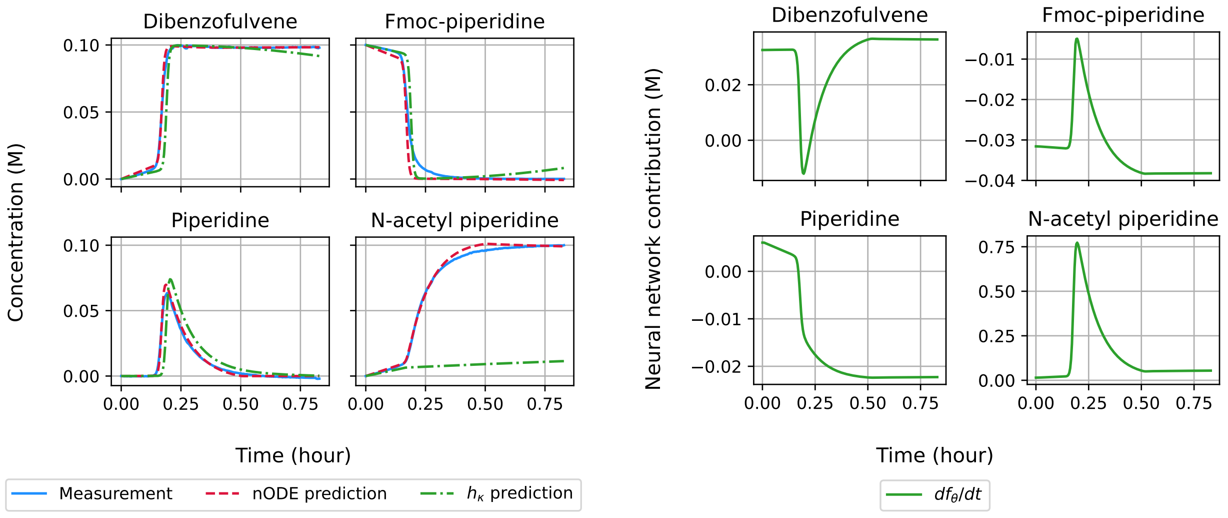

(a) (b)

Figure S3: Predictive performance of the nODE with an incomplete vector field on the single-pulse data in the open system. (a), The experimental measurements, predictions (solid blue) from the nODE (dashed red) and h_κ (dashdotted green). The four panes illustrate the molar concentrations for the species dibenzofulvene, Fmoc-piperidine, piperidine, and N-acetyl piperidine. (b), The neural network contribution for the four measured species.

Sustained Oscillations

Standard Fit

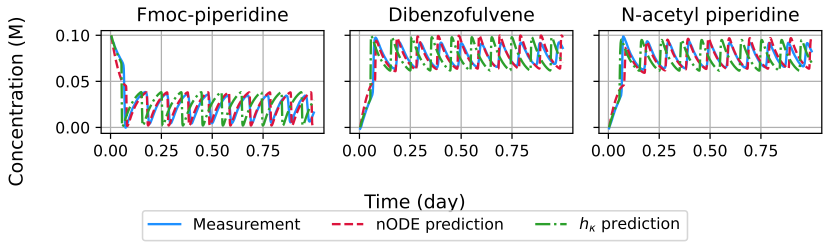

(a)

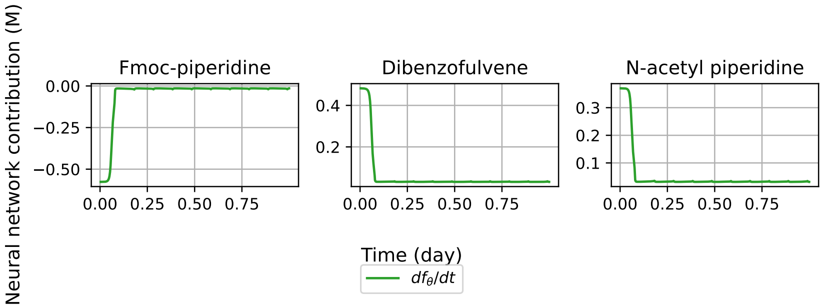

(b)

Figure S4: Predictive performance of the nODE on the oscillating data. (a), The experimental measurements, predictions (solid blue) from the nODE (dashed red) and h_κ (dashdotted green). The three panes illustrate the molar concentrations for the species Fmoc-piperidine, dibenzofulvene, and N-acetyl piperidine. (b), The neural network contribution for the four measured species.

Open System

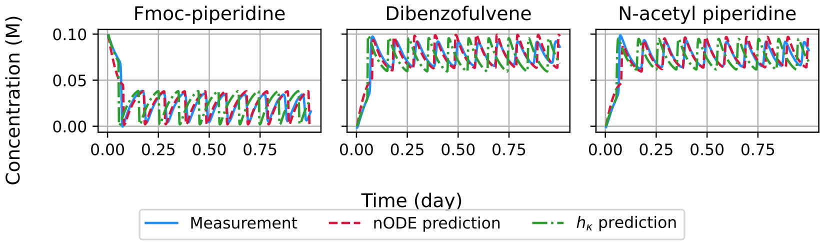

(a)

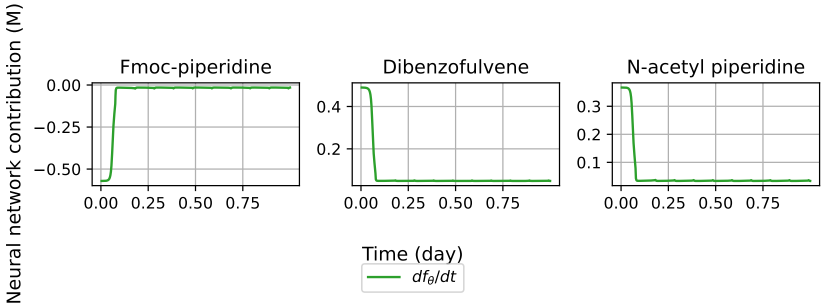

(b)

Figure S5: Predictive performance of the nODE on the oscillating data in the open system. (a), The experimental measurements, predictions (solid blue) from the nODE (dashed red) and h_κ (dashdotted green). The three panes illustrate the molar concentrations for the species Fmoc-piperidine, dibenzofulvene, and N-acetyl piperidine. (b), The neural network contribution for the four measured species.

Missing Reactions

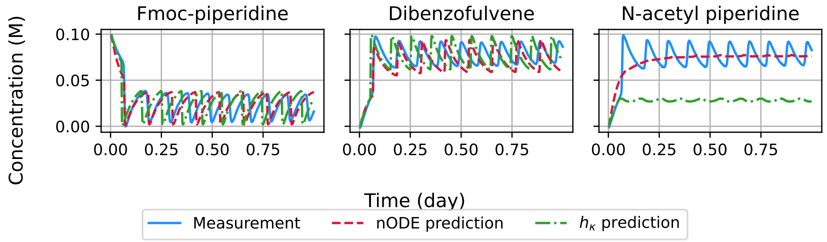

(a)

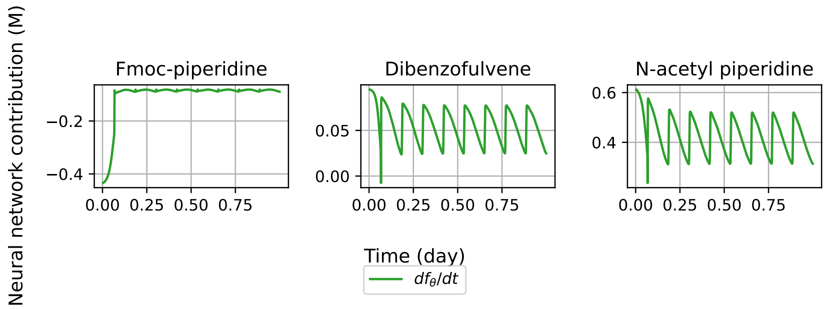

(b)

Figure S6: Predictive performance of the nODE on the oscillating data of the missing reactions dataset. (a), The experimental measurements, predictions (solid blue) from the nODE (dashed red) and h_κ (dashdotted green). The three panes illustrate the molar concentrations for the species Fmoc-piperidine, dibenzofulvene, and N-acetyl piperidine. (b), The neural network contribution for the four measured species.

References

(S1) Bishop, C. M. *Pattern Recognition and Machine Learning*; Springer New York, NY, 2006.

(S2) Hochreiter, S.; Schmidhuber, J. Long short-term memory. *Neural computation* **1997**, *9*, 1735–1780.

(S3) Angeli, D. A Tutorial on Chemical Reaction Network Dynamics. *European Journal of Control* **2009**, *15*, 398–406.

(S4) Upadhyay, S. K. *Chemical Kinetics and Reaction Dynamics*; Springer: New York, 2006.

(S5) Levine, R. D. *Molecular reaction dynamics*; Cambridge University Press: Cambridge, UK, 2009.

(S6) Adams, R. A.; Essex, C. *Calculus: a complete course*, 8th ed.; Pearson: Ontario, 2013.

(S7) Lu, Y.; Zhong, A.; Li, Q.; Dong, B. Beyond finite layer neural networks: Bridging deep architectures and numerical differential equations. International Conference on Machine Learning. 2018; pp 3276–3285.

(S8) Kidger, P. On Neural Differential Equations. Ph.D. thesis, University of Oxford, 2021.

(S9) Chen, R. T.; Rubanova, Y.; Bettencourt, J.; Duvenaud, D. K. Neural ordinary differential equations. *Advances in Neural Information Processing Systems* **2018**, *31*.

(S10) He, K.; Zhang, X.; Ren, S.; Sun, J. Deep Residual Learning for Image Recognition. Proceedings of the IEEE conference on computer vision and pattern recognition. 2016; pp 770–778.

(S11) Rackauckas, C.; Ma, Y.; Martensen, J.; Warner, C.; Zubov, K.; Supekar, R.; Skinner, D.; Ramadhan, A.; Edelman, A. Universal Differential Equations for Scientific Machine Learning. 2021; `https://arxiv.org/abs/2001.04385`.

(S12) Aro, C. J. CHEMSODE: a stiff ODE solver for the equations of chemical kinetics. *Computer Physics Communications* **1996**, *97*, 304–314.

(S13) Ji, W.; Deng, S. Autonomous Discovery of Unknown Reaction Pathways From Data by Chemical Reaction Neural Network. *The Journal of Physical Chemistry A* **2021**, *125*, 1082–1092.

(S14) Alexander, R. Diagonally implicit Runge–Kutta methods for stiff ODE's. *SIAM Journal on Numerical Analysis* **1977**, *14*, 1006–1021.
